# The Am. Gynecological Society

**Published:** 1881-10

**Authors:** 


THE AM. GYNECOLOGICAL SOCIETY met September 22, at the hall of the Academy of Medicine, and listened to the annual address of the President, Dr. W. H. Byford, of Chicago, and to papers by Dr. E. Van de Warker, of Syracuse, and Dr. I. E. Taylor, of New York. These papers and the address occupied the morning session, and in the afternoon there was a discussion on papers read by Willian Goodell, of Philadelphia, and H. F. Campbell, of Augusta, Ga. In the discussion Drs. Baker, Kimball, Dunlap, Sims, Garrigues, Wilson, Reamy, Mary Putnam Jacobi, and others participated.

